# Integrating Network Pharmacology, Molecular Docking, and Experimental Validation: Andrographolide Attenuates Acute Liver Injury via the NLRP3/Caspase-1/GSDMD-Mediated Pyroptosis Pathway

**DOI:** 10.3390/biom15121743

**Published:** 2025-12-16

**Authors:** Yankun Zhang, Shuanghui Liu, Xiaoxia Liang, Lizi Yin, Changliang He

**Affiliations:** 1 Department of Clinical Veterinary, College of Veterinary Medicine, Sichuan Agricultural University, Chengdu 611130, China; 2023203020@stu.sicau.edu.cn (Y.Z.); 2022203015@stu.sicau.edu.cn (S.L.); 2 Natural Medicine Research Center, College of Veterinary Medicine, Sichuan Agricultural University, Chengdu 611130, China; liangxiaoxia@sicau.edu.cn (X.L.); y; 3 Department of Pharmacy, College of Veterinary Medicine, Sichuan Agricultural University, Chengdu 611130, China

**Keywords:** pyroptosis, andrographolide, acute liver injury, network pharmacology, molecular docking

## Abstract

Background/Objectives: Andrographolide (Andro), a natural diterpenoid lactone, possesses a wide range of pharmacological properties, including notable anti-inflammatory, antioxidant, antitumor, and immunomodulatory activities. Despite its acknowledged therapeutic promise, the exact protective mechanisms underlying its efficacy against acute liver injury (ALI) are still not fully understood. Consequently, determining the molecular mechanisms through which andrographolide alleviates ALI is of substantial scientific and clinical relevance. Methods: Andrographolide’s potential targets and pharmacological mechanisms against liver injury were initially identified using network pharmacology and molecular docking. An acute liver injury (ALI) rat model was induced by intraperitoneal injection of lipopolysaccharide (LPS). The therapeutic efficacy of andrographolide in ALI was evaluated by examining liver histopathology, measuring liver function and oxidative stress markers, and quantifying pro-inflammatory cytokine levels. Meanwhile, the expression of key constituents along the NLRP3/caspase-1/GSDMD signaling axis was quantified using RT-qPCR and Western blotting. In parallel, the protective effect of andrographolide via the canonical NLRP3/caspase-1/GSDMD pyroptosis pathway was further examined in vitro using LPS-plus-ATP-stimulated rat hepatocyte BRL-3A cells. Results: Network pharmacology analysis predicted that andrographolide (Andro) protects against liver injury mainly by targeting core regulators of pyroptosis. Molecular docking simulations further indicated stable binding interactions between Andro and key proteins involved in the pyroptotic pathway, such as NLRP3, ASC, GSDMD, and CASP1. These predictions were experimentally confirmed. Andro administration notably mitigated histopathological alterations, restored serum liver function indicators, lowered pro-inflammatory cytokine levels, and alleviated oxidative stress. Importantly, Andro substantially suppressed the expression of critical mediators along the pyroptosis signaling cascade. Conclusions: This study demonstrates that andrographolide (Andro) ameliorates acute liver injury (ALI) by specifically inhibiting the NLRP3/Caspase-1/GSDMD-mediated pyroptosis pathway. By elucidating this underlying molecular mechanism, our work highlights Andro’s potential as a novel and promising therapeutic candidate for ALI.

## 1. Introduction

Acute liver injury (ALI) is a severe pathological process triggered by diverse etiologies, characterized by massive hepatocyte death and rapid deterioration of liver function within a short period [1,2]. Hepatocyte death is primarily triggered by a cascade of events involving mitochondrial dysfunction, oxidative stress, and immune-inflammatory responses [3,4]. The global incidence of ALI has been steadily increasing, rendering it a significant worldwide healthcare burden [5,6]. The clinical course of ALI is highly variable, with approximately 23% of patients progressing to acute liver failure (ALF), death, or requiring liver transplantation [7,8]. This underscores the critical importance of early identification of disease targets and timely intervention, which can effectively halt disease progression and improve outcomes in ALI [9]. Advances in critical care and the application of liver transplantation have raised the survival rate for ALF from historically around 20% to over 60% at present [10,11,12]. However, liver transplantation is limited by challenges such as donor shortage, high costs, and the necessity for lifelong immunosuppression [13]. Consequently, developing effective targeted therapeutic strategies is of paramount importance for improving the prognosis of ALI.

Pyroptosis is a form of programmed cell death that has garnered significant attention in recent years [14,15]. It is mechanistically defined by pore-forming activity driven by gasdermin (GSDM) family proteins [16]. Distinct from apoptosis and necrosis, pyroptosis is primarily characterized by plasma membrane perforation and rupture, leading to the massive release of pro-inflammatory factors and consequently triggering intense inflammatory cascades [17,18]. Unlike the relatively ‘silent’ demise in apoptosis, pyroptosis is often described as an ‘explosive’ and highly inflammatory cell death process [19]. With advancing research, the critical role of pyroptosis in liver diseases is increasingly being uncovered. Particularly in acute liver injury (ALI), pyroptosis actively contributes to hepatocyte destruction and amplifies the local inflammatory response, thereby establishing a vicious cycle that exacerbates hepatic damage [20,21]. Consequently, the targeted development of effective agents to suppress pyroptosis represents a promising therapeutic strategy.

Andrographolide, the primary active constituent derived from the medicinal herb Andrographis paniculata, is a diterpenoid lactone compound [22]. Extensive modern pharmacological studies have revealed that andrographolide possesses a wide range of bioactivities, including anti-inflammatory, antineoplastic, antiviral, and neuroprotective effects [23,24,25]. Notably, multiple seminal studies indicate that andrographolide exerts precise modulation over pyroptosis by targeting multiple nodal points, such as inflammasome activation, caspase activity, and GSDMD (Gasdermin D) protein processing [26,27,28,29]. In the present study, we employed an integrated strategy combining network pharmacology and molecular docking to identify relevant targets, conducted pharmacodynamic evaluations of its efficacy against ALI, and further validated the underlying mechanisms through both in vivo and in vitro experiments, thereby providing a novel direction for subsequent drug development.

## 2. Materials and Methods

### 2.1. Reagents and Antibodies

Andrographolide (5508-58-7) was sourced from Shanghai Yuanye Sci-Bio Technology Co., Ltd. (Shanghai, China); LPS (L2880) was sourced from Sigma-Aldrich (Darmstadt, Germany). Anti-caspase-1 (22915-1-AP), Anti-NLRP3 (19771-1-AP), and Anti-β-tubulin (66240-1-lg) antibodies were obtained from Proteintech (Wuhan, China); Anti-GSDMD (ab219800) antibody was obtained from Abcam (Cambridge, UK); and Anti-ASC (HY-P8107) antibody was obtained from MCE (Belleville, NJ, USA). Enhanced cell counting kit-8 detection kit (C0037) and lactate dehydrogenase (LDH) cytotoxicity detection kit (C0017) were purchased from Beyotime Biotechnology Co., Ltd. (Shanghai, China). Cell apoptosis fluorescence Hoechst 33342/PI double staining kit (CA1120) was purchased from Solebao Technology Co., Ltd. (Beijing, China). Enzyme-linked immunosorbent assay (ELISA) test kit was purchased from Ruixin Biotech (Beijing, China).

### 2.2. Network Pharmacology Methodology

Target data for andrographolide (Andro) were sourced from the Traditional Chinese Medicine Systems Pharmacology (TCMSP) database (https://www.tcmsp-e.com/). Disease-associated targets related to acute liver injury (ALI) were retrieved from the GeneCards database (https://www.genecards.org/) and the OMIM database (https://www.omim.org/). To predict interactions among the targeted genes, the STRING database (https://string-db.org/) was employed, with a confidence threshold of >0.4. All data obtained from these online resources are publicly accessible, and this study strictly adhered to the respective databases’ access policies and publishing guidelines.

Gene Ontology (GO) functional enrichment analysis and Kyoto Encyclopedia of Genes and Genomes (KEGG) pathway enrichment analysis were performed using the WebGestalt analytical platform (http://www.webgestalt.org/) to obtain results. Subsequently, Cytoscape software (version 3.10.1) was employed to calculate topological parameters—including Degree, Closeness, and Betweenness—followed by visualization of the interaction networks.

### 2.3. Molecular Docking Methodology

Core targets for molecular docking were selected from the aforementioned network pharmacology results based on a topological parameter threshold. Specifically, targets exhibiting a Degree value greater than twice the median (Degree = 5) were chosen. These selected protein targets were then imported into the Systems Dock Web Site (https://academic.oup.com/, Version 2.0) server to perform docking validation with andrographolide (AP, i.e., Andro). The binding affinity between AP and each target was assessed according to the corresponding Docking Score.

### 2.4. Animal Experiments and Grouping

Male Wistar rats (200 ± 20 g, SPF grade) were supplied by Beijing Vital River Laboratory Animal Technology Co., Ltd. (Beijing, China). Animals were housed under standard laboratory conditions at the Animal Facility of the College of Veterinary Medicine, Sichuan Agricultural University. After one week of acclimatization under controlled temperature (22–26 °C) and relative humidity (40–60%), with free access to food and water throughout the study. Rats were randomly assigned to five experimental groups (n = 6 per group): Control group, LPS (5 mg/kg) group, LPS + Andro (25 mg/kg) group, LPS + Andro (50 mg/kg) group and LPS + Andro (100 mg/kg) group. Andrographolide (Andro) was administered orally for five consecutive days prior to LPS induction. All animal experimental protocols were reviewed and approved by the Institutional Animal Care and Use Committee of Sichuan Agricultural University (Approval No. 20230045).

### 2.5. Hepatic Index

The hepatic index was calculated based on liver weight and rat body weight using the formula: [Hepatic Index (%) = Liver Weight (g)/Body Weight (g) × 100%].

### 2.6. Hematoxylin-Eosin (H&E) Staining

Liver tissues were fixed in 4% paraformaldehyde, followed by dehydration, paraffin embedding, and sectioning. After deparaffinization with xylene and rehydration through a graded ethanol series, the sections were subjected to H&E staining. Histopathological morphological changes in the rat liver tissues were then examined under an optical microscope.

### 2.7. Immunohistochemistry

After fixation, paraffin embedding, deparaffinization, and antigen retrieval of liver tissue sections, the slides were blocked with 5% BSA for 30 min. Subsequently, they were incubated overnight at 4 °C with primary antibodies against NLRP3, CASPASE-1, GSDMD, and ASC. Next, the sections were treated with goat anti-rabbit IgG polymer at room temperature for 30 min. Finally, immunoreactivity was visualized using 3,3′-diaminobenzidine (DAB) with hematoxylin counterstaining. The stained sections were examined under an optical microscope for density assessment, and quantitative analysis was performed using ImageJ software (version 1.52v).

### 2.8. Serum Biochemical Analysis

Serum levels of ALT, AST, ALP, and γ-GT were measured using a fully automated biochemical analyzer (Hitachi 7180, Tokyo, Japan) according to the manufacturer’s instructions provided with commercial detection kits (CH0101201, Chengdu, China).

### 2.9. Oxidative Stress Assessment

Rat liver tissues were homogenized and centrifuged to collect the supernatants. Using commercially available assay kits (Solarbio, Beijing, China) for MDA, SOD, CAT, and T-AOC, measurements were conducted in accordance with the manufacturers’ protocols to determine the levels of these indicators in both liver homogenates and serum samples.

### 2.10. Enzyme-Linked Immunosorbent Assay

Serum samples, liver tissue homogenates, and cell culture supernatants were collected from the rats. Following the manufacturer’s instructions provided with the respective ELISA kits (Ruixin Biotechnology Co., Ltd., Quanzhou, China), the levels of IL-18, IL-1β, TNF-α, IL-6, and ROS were measured using 50 μL of each sample. The optical density values were analyzed with a microplate reader, and the expression levels were quantified accordingly.

### 2.11. Cell Culture

The BRL-3A rat hepatocyte line was obtained from Immocell Biotechnology Co., Ltd. (IMMOCELL, Cat# IM-R039, Xiamen, China). Cells were cultured in complete high-glucose DMEM medium supplemented with 15% Australian fetal bovine serum (NEWZERUM), 100 U/mL penicillin, and 0.1 mg/mL mg/mL streptomycin. All cultures were maintained at 37 °C in a humidified atmosphere containing 5% CO_2_.

### 2.12. Cell Viability Assay

BRL-3A cells were seeded into 96-well plates and allowed to grow until approximately 80% confluence before stimulation. The experiment consisted of five groups: control group, LPS group, and andrographolide treatment groups (6.25, 12.5, and 25 μg/mL). Each group contained five replicates, and three independent parallel experiments were performed. After the stimulation period, procedures were carried out according to the manufacturer’s instructions of the Enhanced CCK-8 Detection Kit (Beyotime, Shanghai, China).

### 2.13. Lactate Dehydrogenase Release Assay

BRL-3A cells were seeded into 96-well plates and stimulated upon reaching approximately 80% confluency. Cell treatments followed the aforementioned protocol, with additional groups included: a DMEM blank control (no cells) and an LDH maximum release positive control. Upon completion of the stimulation period, the procedure was conducted in strict adherence to the manufacturer’s guidelines of the Lactate Dehydrogenase (LDH) Cytotoxicity Assay Kit (Beyotime, Shanghai, China).

### 2.14. Hoechst 33342/PI Double Staining

BRL-3A cells were seeded onto coverslips placed in 12-well plates (2.5 × 10^5^ cells per well) and stimulated upon reaching approximately 80% confluency. Staining was performed strictly according to the kit protocol. After staining, coverslips were mounted onto glass slides with one drop of anti-fade mounting medium and subsequently observed under a fluorescence microscope. Live cells appeared green in the fluorescent field, while dead cells were visualized in red.

### 2.15. Flow Cytometry

Single-cell suspensions were prepared at a concentration of 10^6^ cells/mL. A 100 µL aliquot of the suspension was centrifuged at 300× *g* for 5 min, after which the supernatant was discarded. The pellet was resuspended in 100 µL of freshly prepared 1× binding buffer, followed by incubation with 5 µL of Annexin V-FITC dye for 10 min at room temperature while protected from light. Then, 10 µL of propidium iodide (PI) solution was added, followed by another 5 min incubation under light-protected conditions. Subsequently, 400 µL of PBS was added to resuspend the cells prior to immediate acquisition. Analysis was performed using a flow cytometer (CytoFLEX, Beckman Coulter, Brea, CA, USA) for data collection and subsequent analysis.

### 2.16. Western Blotting

Proteins were extracted from rat liver tissues. Protein concentrations were determined using a BCA Protein Assay Kit (Beyotime Biotechnology Co., Ltd., Shanghai, China). Equal amounts of proteins were separated by SDS-PAGE electrophoresis (80 V for 30 min in stacking gel, 120 V for 1 h in separating gel). Proteins were then transferred to PVDF membranes using wet transfer method (120 V, 200 mA, 35 min). The membranes were blocked with 5% non-fat milk for 1 h, followed by overnight incubation at 4 °C with the following primary antibodies: caspase-1 (1:2000 dilution), NLRP3 (1:1000 dilution), ASC (1:1000 dilution), GSDMD (1:2000 dilution), and Tubulin (1:5000 dilution). After washing three times with TBST (5 min each), the membranes were incubated with secondary antibody (1:10,000 dilution) at room temperature for 1 h. After three additional TBST washes (5 min each), the membranes were incubated with ECL substrate for 1 min and imaged in a darkroom. Band intensities were quantified using ImageJ software. Results are presented as ratios of band intensity in each group relative to the control group. Original figures can be found in Supplementary Materials.

### 2.17. Real-Time Quantitative Reverse Transcription PCR

Total RNA was extracted from cultured cells using TRIzol reagent (Servicebio, Wuhan, China). Reverse transcription was performed using the SweScript All-in-One RT SuperMix for qPCR (Servicebio). Quantitative real-time PCR was performed on a StepOnePlus instrument (Analytik Jena, Jena, Germany) using SYBR Green Premix (Servicebio). The relative mRNA expression of target genes was calculated using the 2^−ΔΔCt^ method. Primers used for qPCR were purchased from Servicebio and validated prior to use. Primer sequences are listed in Table 1.

### 2.18. Data Analysis

Experimental data were statistically analyzed using SPSS 22.0 and are presented as mean ± standard deviation (Mean ± SD). Intergroup comparisons were performed by ANOVA to assess statistical significance, with *p* < 0.05 considered statistically significant. All histograms in this study were generated using GraphPad Prism 8.0 software.

## 3. Results

### 3.1. Network Pharmacology-Based Mechanistic Insights into Andrographolide Intervention in Acute Liver Injury

**Figure 1:**
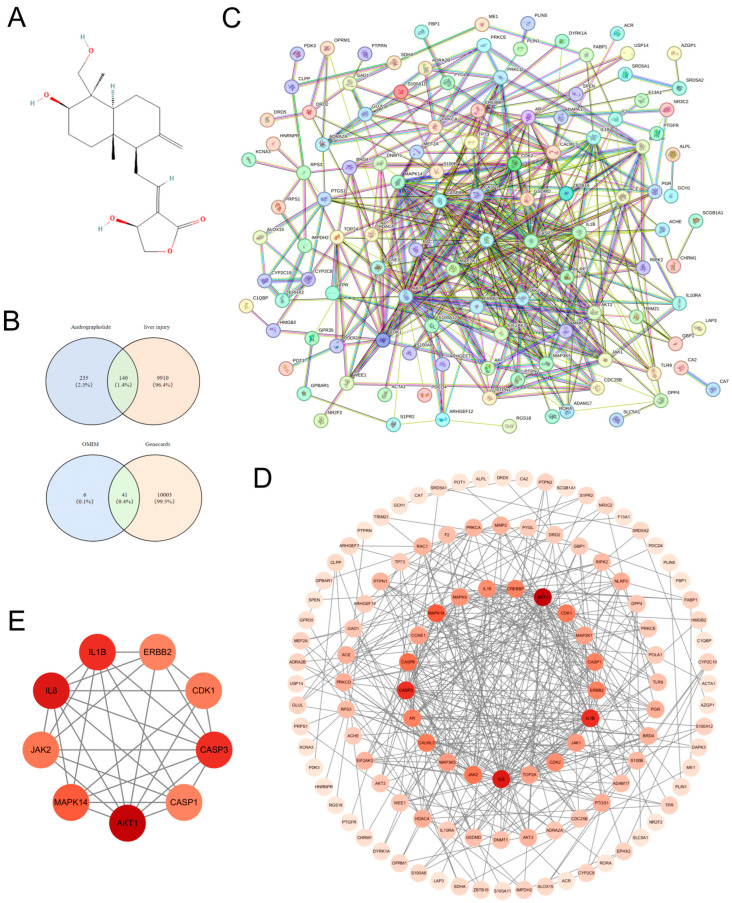
Network Pharmacology-Based Study on the Mechanism of Andrographolide Intervention in Acute Liver Injury. (**A**) Two-dimensional chemical structure of andrographolide. (**B**) Venn diagram illustrating the overlap between andrographolide targets and acute liver injury disease targets. The intersection reveals 140 shared common targets. (**C**) Protein–protein interaction (PPI) network of the common targets. (**D**) Visualization of topological analysis of the PPI network. (**E**) Top 9 core targets identified through network topology analysis.

As shown in Figure 1, network pharmacology results indicate that the common targets of andrographolide and liver injury are closely linked to pyroptosis. Figure 1A displays the 2D structure of andrographolide. The intersection between andrographolide and acute liver injury targets was visualized in a Venn diagram (Figure 1B), identifying 140 shared targets as potential direct sites for andrographolide’s intervention in acute liver injury. These common targets were imported into the STRING database to construct a PPI network, which was further analyzed topologically using Cytoscape (Figure 1C). Visual analysis of the PPI network (Figure 1D) ultimately screened the top 9 core targets, including AKT1, IL6, IL1B, CASP3, MAPK14, CASP1, CDK1, JAK2, and ERBB2 (Figure 1E). Notably, key regulatory molecules in the pyroptosis pathway—such as IL1B, CASP3, and CASP1—were among these core targets, preliminarily indicating that andrographolide’s mechanism may be intimately connected with pyroptosis progression.

**Figure 2:**
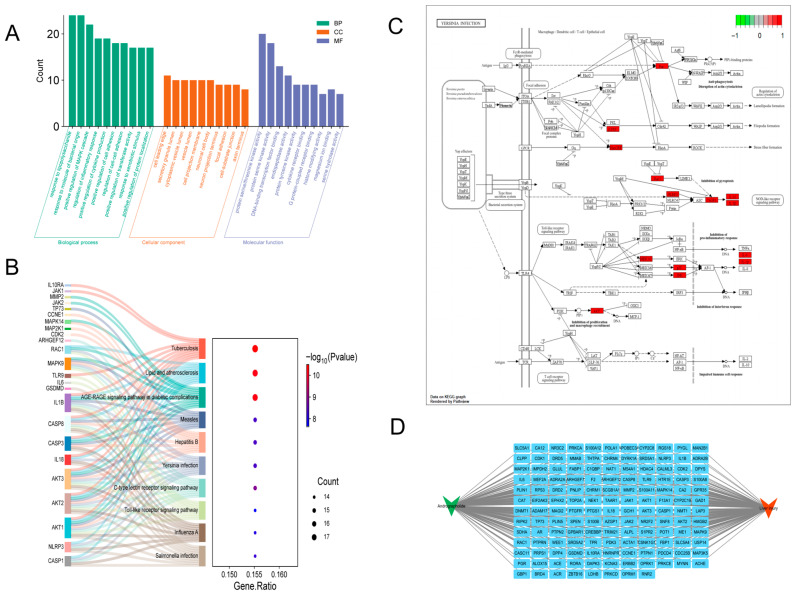
Systematic Identification of Therapeutic Targets and Mechanisms of Andrographolide against Acute Liver Injury. (**A**) Bar plot of GO functional enrichment analysis for the common targets, showing the most significantly enriched terms in Biological Process (BP), Cellular Component (CC), and Molecular Function (MF). (**B**) Bubble chart of KEGG pathway enrichment analysis for the common targets. (**C**) Diagram of the *Yersinia* infection pathway, where targets potentially modulated by andrographolide are highlighted in red. (**D**) Network diagram of “Andrographolide–Acute Liver Injury–Core Targets”, intuitively depicting the synergistic multi-target regulatory mode.

To systematically elucidate the mechanism of action of andrographolide, we performed GO functional annotation and KEGG pathway enrichment analyses on the common targets. The GO analysis results (Figure 2A) reveal that the biological functions of the andrographolide–liver injury interaction are primarily enriched in the following Biological Processes: response to lipopolysaccharide, response to molecule of bacterial origin, positive regulation of MAPK cascade, regulation of inflammatory response, and positive regulation of cytokine production. These functions are all highly associated with hepatocyte stress injury and programmed cell death processes. Importantly, KEGG pathway enrichment analysis (Figure 2B) indicates that the common targets are significantly enriched in multiple signaling pathways related to inflammation and cell death. Among them, the Yersinia infection pathway (Figure 2C) is notably enriched and ranks among the top pathways. Additionally, Hepatitis B, Toll-like receptor signaling pathway, and Salmonella infection represent not only core pathological pathways in acute liver injury but also key upstream regulatory pathways of pyroptosis. These analytical results strongly suggest that andrographolide may exert its hepatoprotective effects by intervening in the interactive network of these pathways, thereby precisely regulating the pyroptosis process in hepatocytes, constituting one of its central protective mechanisms.

To visually illustrate the specific relationship between andrographolide and the pyroptosis pathway, we extracted 24 core targets and performed KEGG pathway enrichment analysis using the Metascape database under parameters identical to those applied in the GO enrichment analysis. The results indicated that andrographolide exerts its anti-liver injury effects by modulating the Yersinia infection signaling pathway. Based on this finding, an “andrographolide–key liver injury targets” network diagram (Figure 2D) was constructed. This network clearly demonstrates that andrographolide, as a single active molecule, can directly or indirectly act on key proteins in the executive phase of pyroptosis (e.g., GSDMD) and their upstream activating signals (e.g., NLRP3 inflammasome, CASP1), forming a synergistic multi-target regulatory mode for intervening in acute liver injury.

### 3.2. Validation of Binding Affinity Between Andrographolide and Core Targets via Molecular Docking

**Figure 3:**
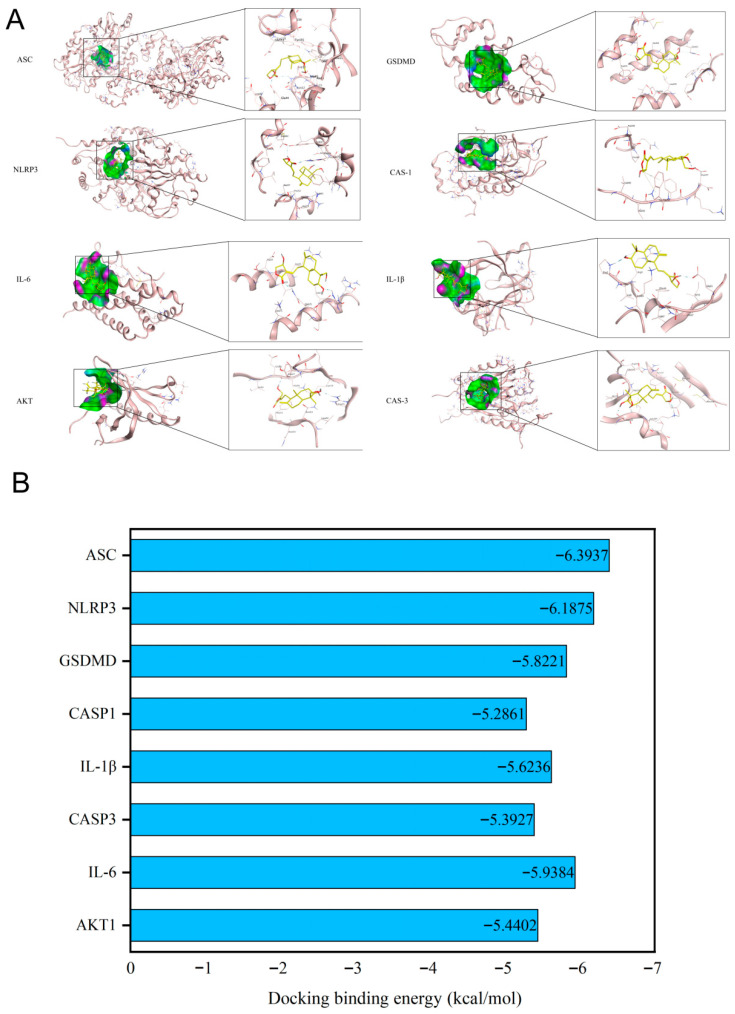
Results of molecular docking validation. (**A**) Three-dimensional binding mode diagrams of andrographolide with core targets (ASC, NLRP3, GSDMD, CASP1, IL-1β, CASP3, IL-6, AKT1). In the figures, andrographolide is represented as a stick model, target proteins are depicted as cartoon models, and hydrogen bonds are indicated by yellow dashed lines. (**B**) Histogram of binding energies between andrographolide and individual core targets.

To validate the interactions between andrographolide and the core targets of acute liver injury predicted by network pharmacology and further elucidate its mechanism of action, we selected key molecules involved in the pyroptosis pathway and related signaling pathways (ASC, NLRP3, GSDMD, CASP1, IL-1β, CASP3, IL-6, AKT1) for molecular docking verification. The three-dimensional binding mode analysis revealed (Figure 3A) that Andrographolide mainly binds stably within the active pockets of the targets through hydrogen bonding, hydrophobic interactions, and π-π stacking. Collectively, these interactions stabilize the compound–target complexes and provide a structural basis for their functional modulation.

Binding energy analysis results revealed (Figure 3B): Andrographolide formed strong interactions with all selected core targets, with binding energies lower than −5.0 kcal/mol (Panel B). Among them, the lowest binding energy was observed with ASC (−6.3937 kcal/mol), indicating the strongest affinity, followed by NLRP3 (−6.1875 kcal/mol), IL-6 (−5.9384 kcal/mol), GSDMD (−5.8221 kcal/mol), IL-1β (−5.6236 kcal/mol), AKT1 (−5.4402 kcal/mol), CASP3 (−5.3927 kcal/mol), and CASP1 (−5.2861 kcal/mol). The binding energies of all targets met the threshold for strong binding (<−5.0 kcal/mol), confirming the high affinity between andrographolide and these targets.

### 3.3. Protective Effect of Andrographolide Against Lipopolysaccharide-Induced Acute Liver Injury in Rats

**Figure 4:**
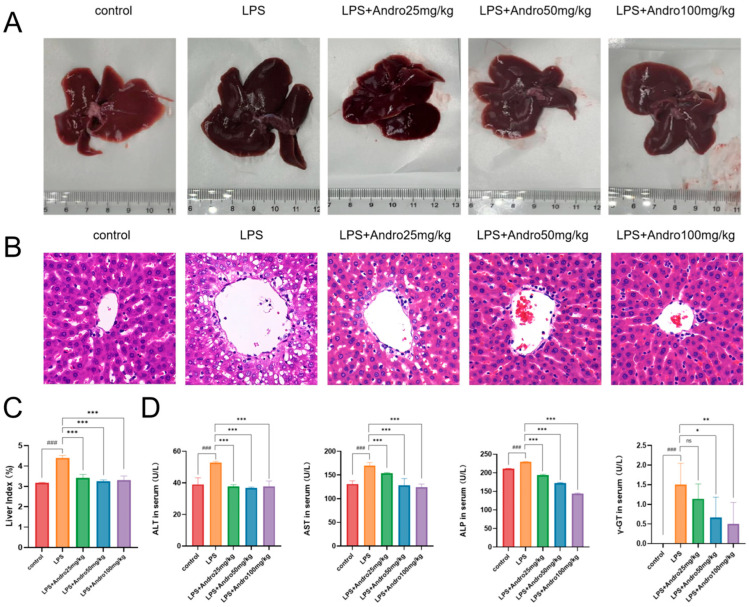
Protective effect of andrographolide against LPS-induced acute liver injury in rats. (**A**) Representative macroscopic views of liver tissue from each experimental group. (**B**) Hematoxylin and eosin (H&E)-stained sections of liver tissue (40× magnification). (**C**) Statistical histogram of liver indices. (**D**) Serum ALT, AST, ALP, and γ-GT levels across different groups. Data are presented as mean ± SD (n = 6). ### *p* < 0.001 versus Control group; * *p* < 0.05, ** *p* < 0.01, *** *p* < 0.001 versus LPS group; ns, not statistically significant.

To evaluate the therapeutic efficacy of andrographolide in ameliorating liver injury, a rat model of drug-induced liver injury was established. Results demonstrated that andrographolide effectively improved morphological damage, pathological injury, liver index, and serum biochemical markers in rats with liver injury, thereby alleviating the condition. Macroscopic observation of the livers (Figure 4A) showed that compared to the control group, the LPS model group exhibited significantly darker liver color (dark purple) with scattered hemorrhagic spots and ecchymoses on the surface, suggesting severe congestion, swelling, and ischemic injury. In contrast, the andrographolide-treated groups (25, 50, 100 mg/kg) showed dose-dependent improvements in liver morphology, approaching the appearance of the control group. Histopathological examination by H&E staining (Figure 4B) revealed that the LPS model group, compared to the control, displayed hepatocellular degeneration and necrosis (characterized by cytoplasmic vacuolation and ballooning degeneration), along with pyknotic and fragmented nuclei in some hepatocytes, sinusoidal dilation and congestion, and substantial inflammatory cell infiltration (including neutrophils and macrophages) in the portal areas and hepatic parenchyma. These pathological changes were effectively ameliorated by andrographolide treatment. As shown in Figure 4C, the liver index was significantly elevated in the LPS model group, indicating marked hepatic swelling due to inflammatory responses. The andrographolide-treated groups exhibited a dose-dependent reduction in the liver index, bringing it close to the level observed in the control group. This suggests that medium and high doses of andrographolide effectively inhibited liver swelling. Serum biochemical indicators (ALT, AST, ALP, γ-GT) are classical markers reflecting hepatocellular and biliary tract damage. Measurement results (Figure 4D) indicated that andrographolide treatment led to a dose-dependent decrease in these serum biochemical parameters, effectively mitigating the abnormal blood biochemical profiles associated with liver injury.

### 3.4. Regulatory Effects of Andrographolide on Oxidative Stress and Inflammatory Response in LPS-Induced Acute Liver Injury in Rats

**Figure 5:**
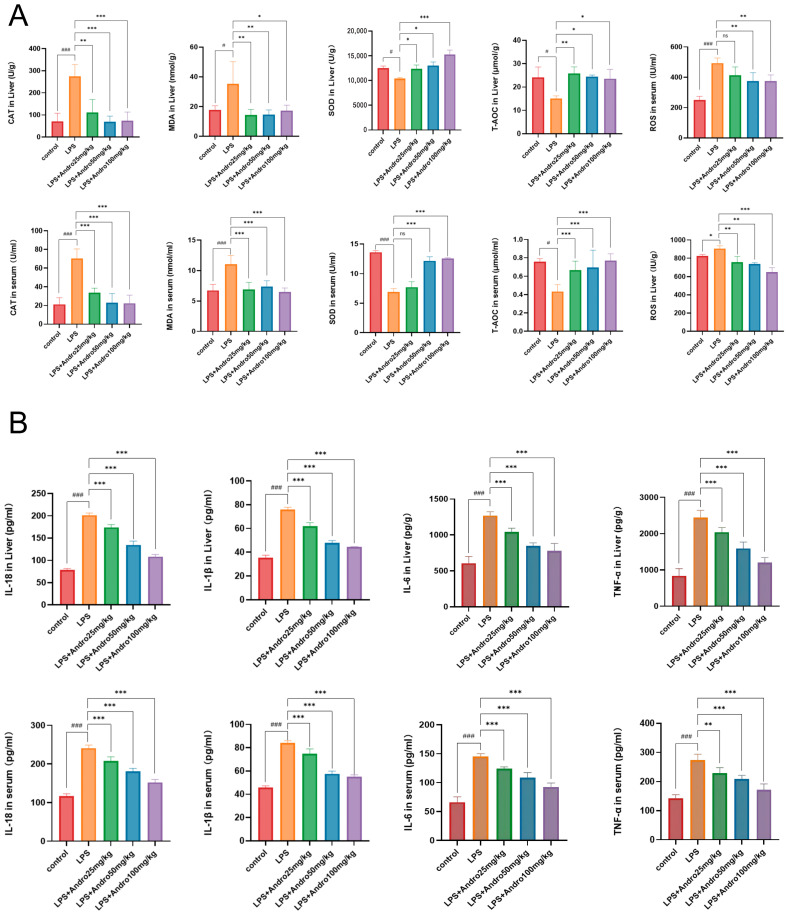
Regulatory effects of andrographolide on oxidative stress and inflammation in rats with LPS-induced acute liver injury. (**A**) Quantification of oxidative stress-related markers in liver tissue and serum. Antioxidant indicators include CAT (catalase), SOD (superoxide dismutase), and T-AOC (total antioxidant capacity); oxidative damage markers include MDA (malondialdehyde) and ROS (reactive oxygen species). (**B**) Levels of inflammatory cytokines in liver tissue and serum. Measured analytes comprise IL-18, IL-1β, IL-6, and TNF-α (all being pro-inflammatory cytokines). Data are expressed as mean ± SD (n = 4 per group). # *p* < 0.05, ### *p* < 0.001 vs. Control group; * *p* < 0.05, ** *p* < 0.01, *** *p* < 0.001 vs. LPS group; ns denotes no statistical significance (*p* ≥ 0.05).

To further clarify the molecular mechanism by which andrographolide ameliorates LPS-induced acute liver injury, we measured oxidative stress-related markers (Figure 5A) and pro-inflammatory cytokine levels (Figure 5B) in both liver tissue and serum. The results demonstrate that its protective effect is closely associated with the suppression of oxidative stress imbalance and attenuation of the inflammatory response. As shown in Figure 5A, both in liver tissue and serum, SOD activity and total antioxidant capacity (T-AOC) were significantly decreased in the LPS model group compared to the control group (### *p* < 0.001), whereas catalase (CAT) activity, malondialdehyde (MDA, a marker of lipid peroxidation) content, and reactive oxygen species (ROS) levels were markedly increased (### *p* < 0.001), indicating that LPS induced severe oxidative stress injury. Treatment with andrographolide resulted in a dose-dependent improvement in the oxidative stress status. The results in Figure 5B show that in both liver tissue and serum, the levels of IL-1β, IL-6, TNF-α, and IL-18 were significantly higher in the LPS model group than in the control group (### *p* < 0.001), suggesting a robust inflammatory response. Conversely, andrographolide administration produced a dose-dependent suppression of pro-inflammatory cytokine secretion. These findings align with the previously described trends toward histological and biochemical improvement, providing further confirmation that the hepatoprotective role of andrographolide against LPS-induced acute liver injury is likely mediated through a dual mechanism—inhibiting oxidative stress imbalance and attenuating the inflammatory response.

### 3.5. Andrographolide Suppresses Pyroptosis in Rats with LPS-Induced Acute Liver Injury

**Figure 6:**
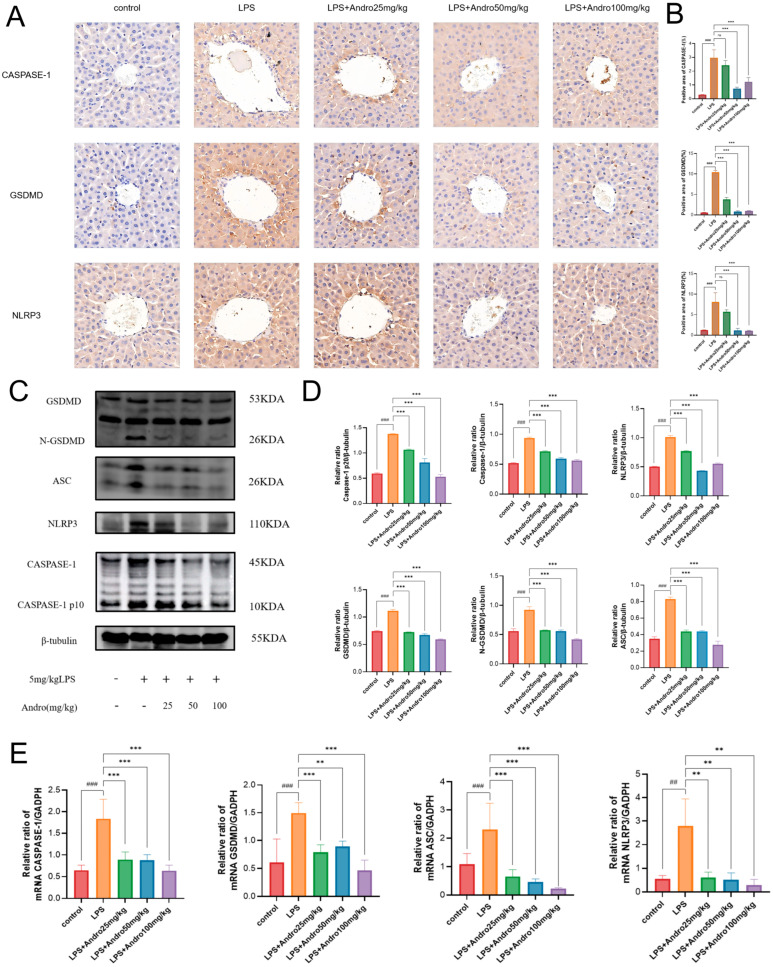
Inhibitory effect of andrographolide on hepatocyte pyroptosis in rats with LPS-induced acute liver injury. (**A**) Representative immunohistochemistry images showing NLRP3, Caspase-1, and GSDMD expression in liver tissue (40× magnification). (**B**) Quantitative analysis of immunohistochemically positive area. (**C**) Representative Western blot bands of key proteins in the pyroptosis pathway. From top to bottom: NLRP3, ASC, Pro-caspase-1, Cleaved-caspase-1 (p10), GSDMD, and N-GSDMD. β-Tubulin was used as the internal loading control. (**D**) Grayscale value quantification of Western blot results. (**E**) mRNA expression levels of key genes in the pyroptosis pathway detected by RT-qPCR. All data are expressed as mean ± SD (n = 3). ## *p* < 0.01, ### *p* < 0.001 vs. Control group; ** *p* < 0.01, *** *p* < 0.001 vs. LPS group; ns, not statistically significant.

Immunohistochemical results (Figure 6A) demonstrated significantly enhanced positive staining for CASPASE-1, GSDMD, and NLRP3 in the LPS model group, characterized by dense brownish-yellow granules and intensified coloration in the cytoplasm, indicating widespread activation of pyroptosis. According to the quantification of immunohistochemically positive area (Figure 6B), andrographolide treatment dose-dependently suppressed the expression of pyroptosis-related factors. Western blot analysis (Figure 6C,D) revealed that andrographolide inhibits NLRP3 inflammasome assembly, Caspase-1 activation, and GSDMD cleavage, thereby blocking the execution of pyroptosis. Real-time quantitative PCR results (Figure 6E) showed that the mRNA levels of pyroptosis-related genes (Caspase-1, GSDMD, ASC, NLRP3) in the liver tissue of the LPS model group were markedly upregulated compared to the control group, suggesting transcriptional-level activation of the pyroptosis pathway by LPS. Andrographolide treatment significantly reduced the mRNA levels of these genes. Collectively, these findings elucidate the core molecular mechanism by which andrographolide ameliorates acute liver injury: by suppressing the NLRP3/Caspase-1/GSDMD-mediated pyroptosis pathway, reducing hepatocyte pyroptosis and subsequent inflammatory responses to exert its hepatoprotective effects.

### 3.6. Andrographolide Ameliorates LPS-Induced Injury in BRL-3A HepatA Hepatocytes In Vitro

Our preceding in vivo experiments confirmed the overall hepatoprotective effect of andrographolide against acute liver injury. However, given the complexity of the intact organism, whether such protection stems from direct actions on parenchymal hepatocytes or indirect mechanisms involving modulation of the immune system remains unclear. To verify the direct cytoprotective impact of andrographolide on liver cells while excluding interference from systemic neurohumoral factors, we established an LPS-induced injury model using BRL-3A cells for in vitro pharmacodynamic evaluation.

**Figure 7:**
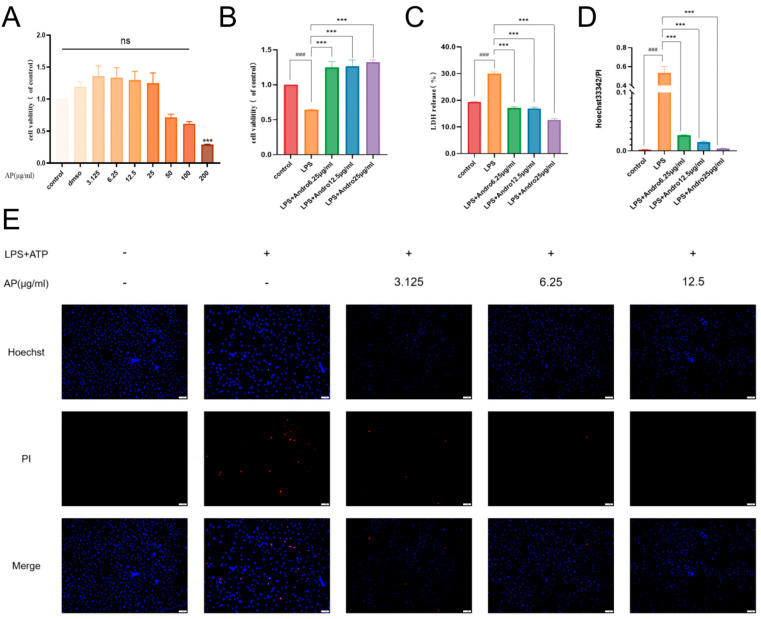
Andrographolide attenuates LPS-induced injury in BRL-3A hepatocytes in vitro. (**A**) Effect of andrographolide on BRL-3A cell viability. Cell viability was measured after treatment with varying concentrations of andrographolide (0, 3.125, 6.25, 12.5, 25, 50 µg/mL) for 12 h. (**B**) Improvement of LPS-induced loss of BRL-3A cell viability by andrographolide (CCK-8 assay). Cells were pretreated with andrographolide (6.25, 12.5, 25 µg/mL) for 16 h, then exposed to LPS (0.1 mg/mL) for 6 h before measuring cell viability. (**C**) Inhibitory effect of andrographolide on LPS-induced membrane damage in BRL-3A cells (LDH release assay). (**D**) Reduction in LPS-induced BRL-3A cell death by andrographolide (quantified by PI double staining). (**E**) Fluorescence micrographs (scale bar, 20 μm) showing suppression of LPS-induced BRL-3A cell death (Hoechst/PI double staining). Under fluorescence microscopy, nuclei in the Control group appeared uniformly blue (Hoechst staining) without red fluorescence (PI staining, dead cells). All data are presented as mean ± SD (n = 5). ### *p* < 0.001 versus Control group; *** *p* < 0.001 versus LPS model group; “ns” indicates no statistical significance (*p* > 0.05).

To ensure that any observed effects were not confounded by inherent cytotoxicity of the compound, we first assessed the influence of various concentrations of andrographolide (0, 3.125, 6.25, 12.5, 25, 50 μg/mL) on the viability of BRL-3A cells via the CCK-8 assay (Figure 7A). The results indicated that concentrations ranging from 0 to 25 μg/mL did not significantly inhibit cell viability (*p* > 0.05 vs. Control group). Accordingly, low (6.25 μg/mL), medium (12.5 μg/mL), and high (25 μg/mL) doses were selected for follow-up experiments. After inducing injury in BRL-3A cells with LPS (0.1 mg/mL) plus ATP, CCK-8 assay was again employed to measure cell viability (Figure 7B). The data demonstrate that andrographolide directly promotes the survival of injured hepatocytes. Lactate dehydrogenase release, a classic indicator of plasma membrane integrity, was also measured (Figure 7C). The results showed that andrographolide effectively stabilizes membrane structure and mitigates LPS-induced membrane damage. Cell death was quantified by Hoechst 33342/PI double staining (Figure 7D,E). In the LPS model group, the rate of PI-positive cells rose markedly to approximately 45% (### *p* < 0.001 vs. Control group), with fluorescence microscopy revealing numerous red-fluorescent cells (Figure 7E). In contrast, the high-dose andrographolide group showed nearly complete disappearance of red fluorescence (Figure 7E), corroborating that andrographolide significantly suppresses LPS-induced hepatocyte death.

### 3.7. Inhibition of Pyroptosis by Andrographolide in BRL-3A Cells In Vitro

**Figure 8:**
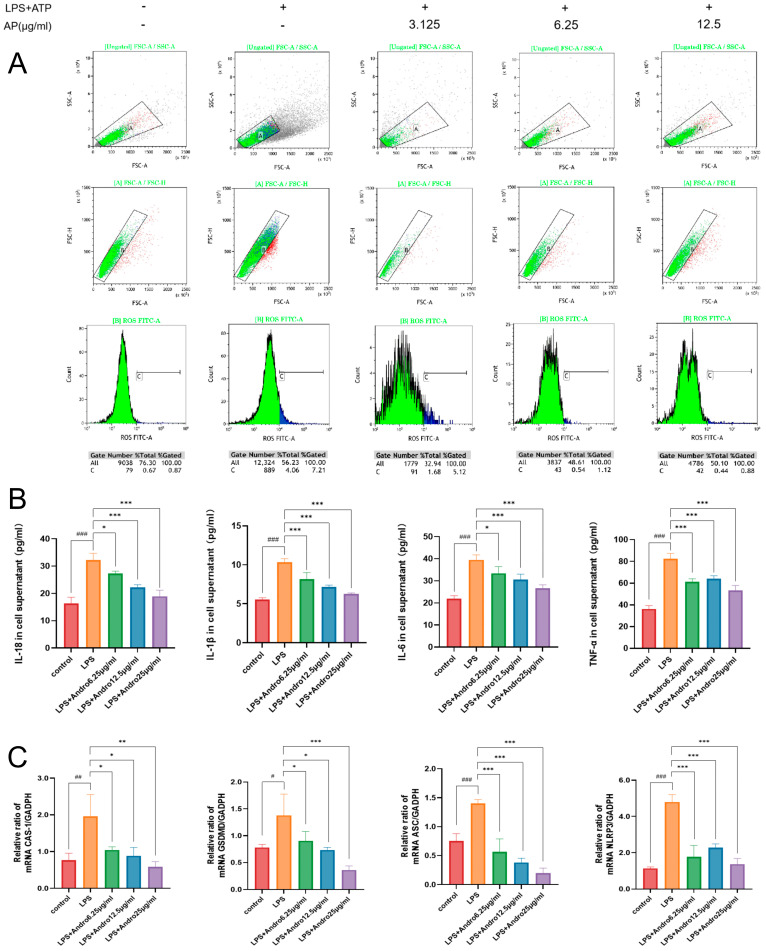
Andrographolide suppresses LPS-induced pyroptosis in BRL-3A cells in vitro. (**A**) Effect of andrographolide (Andro) on LPS-induced ROS production in BRL-3A cells, measured by flow cytometry. (**B**) Effect of Andro on the secretion of inflammatory factors in LPS-induced BRL-3A cells, determined by ELISA. (**C**) Effect of Andro on the expression of pyroptosis-related genes in LPS-induced BRL-3A cells, analyzed by qPCR. All data are presented as the mean ± SD (n = 3). # *p* < 0.05, ## *p* < 0.01, ### *p* < 0.001 compared with the Control group; * *p* < 0.05, ** *p* < 0.01, *** *p* < 0.001 compared with the LPS model group.

Pyroptosis is a key pathological process in LPS-induced acute liver injury. While our preliminary in vitro experiments have confirmed the direct protective effect of andrographolide on LPS-injured BRL-3A hepatocytes, we next sought to determine whether this protection is achieved through the suppression of pyroptosis. Reactive oxygen species (ROS) serve as critical upstream signaling molecules for NLRP3 inflammasome activation, and their excessive accumulation can trigger pyroptosis. Flow cytometry analysis of intracellular ROS levels in BRL-3A cells (Figure 8A) demonstrated that andrographolide effectively inhibits LPS-induced ROS overproduction, thereby blocking the upstream trigger signal for pyroptosis. ELISA measurement of inflammatory cytokine levels in the cell supernatant (Figure 8B) revealed that andrographolide significantly suppresses the release of downstream inflammatory factors of pyroptosis, thereby mitigating inflammatory damage. Furthermore, qPCR detection of pyroptosis-related gene expression (NLRP3, ASC, Caspase-1, GSDMD; Figure 8C) showed that andrographolide markedly downregulates LPS-induced expression of NLRP3, ASC, GSDMD, and CASPASE-1, consequently inhibiting pyroptosis and further alleviating liver injury.

## 4. Discussion

The rising global incidence and mortality of Acute Liver Injury (ALI) underscore its status as a critical public health challenge, necessitating the development of targeted therapies for early intervention and effective disease management [30,31,32]. This context makes the exploration of highly effective and low-toxicity hepatoprotective agents from Chinese herbal medicine a particularly promising strategy [33,34]. Andrographolide, the principal bioactive compound derived from Andrographis paniculata, has been pharmacologically validated to exert robust protective effects against diverse etiologies of ALI [35,36,37]. It has been demonstrated to significantly mitigate elevations in serum transaminase levels and improve histopathological damage induced by various hepatotoxins, including acetaminophen (APAP), D-galactosamine (D-GalN), and lipopolysaccharide (LPS) [38,39,40,41]. Despite its well-documented benefits, the precise molecular underpinnings of andrographolide’s hepatoprotective effects remain incompletely understood. The prevailing paradigm has largely attributed these effects to non-specific anti-inflammatory and antioxidant properties. However, such a generalized explanation fails to account for its distinct efficacy within the complex pathophysiology of acute liver injury. Consequently, a systematic elucidation of its specific mode of action is necessary to unlock its full therapeutic potential and guide its clinical translation.

To systematically decipher the multi-target characteristics of andrographolide, we initially employed a network pharmacology approach. Protein–protein interaction (PPI) network analysis (Figure 1C) identified a hub of core targets, including AKT1, IL6, IL1B, CASP3, MAPK14, CASP1, CDK1, JAK2, and ERBB2 (Figure 1E). Importantly, GO and KEGG enrichment analyses (Figure 2A,B) provided crucial clues: these targets were significantly enriched in multiple signaling pathways intimately associated with inflammation and cell death. The prominent enrichment in pathways such as Yersinia infection and other inflammation-related pathways drew our attention to pyroptosis—a form of programmed inflammatory cell death [42,43,44]. This led us to hypothesize that the hepatoprotective effect of andrographolide might be closely linked to its fine-tuned regulation of NLRP3 inflammasome-mediated pyroptosis. The essence of pyroptosis lies in the formation of pores in the cell membrane by Gasdermin family proteins (particularly GSDMD), leading to osmotic lysis and the massive release of pro-inflammatory cytokines such as interleukin-1β (IL-1β) and IL-18 [45,46,47,48]. This process amplifies simple hepatocyte death into a vigorous local or even systemic inflammatory storm. Molecular docking results revealed that andrographolide could stably bind into the active pockets or allosteric sites of several core target proteins (including NLRP3, Caspase-1, and GSDMD) with favorable binding free energies (generally below −5 kcal/mol) (Figure 3B). This high-affinity binding pattern strongly suggests that andrographolide is not merely a common ligand but likely acts as a direct allosteric inhibitor. By binding to specific sites on these proteins, it presumably induces conformational changes that suppress their normal biological functions, providing a structural biology perspective on how it may curtail the initiation of pyroptosis at its source.

To verify whether andrographolide ameliorates liver injury by inhibiting pyroptosis, subsequent in vivo and in vitro experiments were conducted. The results demonstrated that andrographolide effectively improved morphological and pathological liver damage, as well as liver enzyme profiles and other relevant injury markers in animal models. Correspondingly, in cellular models, andrographolide enhanced the viability of BRL-3A cells and reduced cellular damage. Notably, andrographolide markedly mitigated oxidative stress indicators at both the organismal (rat) and cellular levels. A published study established reactive oxygen species (ROS), particularly those of mitochondrial origin, as a pivotal activator of the NLRP3 inflammasome [49,50]. A burst of reactive oxygen species (ROS) can directly act on the NLRP3 protein, triggering its conformational change and oligomerization, which initiates inflammasome assembly [51,52]. Thus, the alleviation of oxidative stress by andrographolide represents a critical upstream event that prevents NLRP3 inflammasome activation. This cascade is likely initiated when andrographolide, through its intrinsic antioxidant property, scavenges excessive ROS and thus disrupts the signaling pathway upstream of NLRP3. Notably, the restorative effect of andrographolide on antioxidant capacity did not follow a strictly linear concentration-dependent relationship. As shown in Figure 4A, even at relatively low concentrations, andrographolide significantly counteracted the LPS-induced depletion of CAT, MDA, and T-AOC. However, further increases in concentration led to a plateau in the extent of improvement, indicating a characteristic saturation effect. Recent studies have revealed that certain natural flavonoids mitigate liver injury via activation of the autophagy–Nrf2 signaling axis—an effect abolished by autophagy inhibitors—suggesting that sufficient Nrf2 activation depends on upstream autophagic processes [53]. A plausible explanation for our observations is that andrographolide, acting as an Nrf2 pathway agonist, efficiently triggers the body’s self-defense mechanisms, such as upregulating antioxidant enzymes, once a certain plasma concentration threshold is reached [54,55]. Beyond this threshold, increasing the drug concentration may not produce substantially greater benefit. Rather than undermining our core findings, this behavior highlights a mode of action distinct from that of single-target inhibitors: instead of strongly inhibiting pathological pathways, andrographolide modulates redox homeostasis through a more balanced regulatory mechanism.

As the assembly of the NLRP3 inflammasome (NLRP3, ASC, pro-caspase-1) is a cornerstone of canonical pyroptosis, we assessed its key components. Our Western blot and qPCR analyses (Figure 6C–E and Figure 7C) confirmed that andrographolide significantly suppresses the expression of these molecules. Intriguingly, this aligns with a reported mechanism wherein andrographolide, by inducing mitophagy via P110 binding, eliminates damaged mitochondria. Given that mitochondrial dysfunction is a primary source of ROS, this previously established activity provides a plausible mechanistic foundation for our current findings: the removal of the upstream trigger (ROS) via mitophagy subsequently abolishes the downstream expression and activation of the inflammasome. By clearing damaged mitochondria, this process removes a critical upstream signal for NLRP3 inflammasome activation, thereby defining the P110–mitophagy axis as the specific route through which andrographolide exerts its inhibitory effect [56]. Furthermore, emerging evidence suggests that andrographolide may suppress NLRP3 inflammasome activation through alternative pathways. For example, in a murine model of gouty arthritis, the compound was found to inhibit the IKK/NF-κB/TXNIP axis, leading to downregulation of NLRP3 and pro-IL-1β expression, ultimately attenuating inflammation and monocyte infiltration [27,57]. This finding aligns with our current results, demonstrating that andrographolide alleviates liver injury by suppressing the NLRP3/Caspase-1/GSDMD-mediated pyroptosis pathway.

## 5. Conclusions

In summary, andrographolide exerts a concerted, dual-phase inhibition on the pyroptotic cascade, thereby underpinning its potent protective effects. Mechanistically, it attenuates the upstream initiation phase by quenching ROS generation and concurrently impedes the downstream execution phase by suppressing NLRP3 inflammasome assembly, GSDMD cleavage, and pro-inflammatory cytokine maturation. This multi-targeted pharmacological strategy likely affords superior efficacy over compounds that modulate a singular pathway component. Together, our data firmly establish andrographolide as a highly promising lead compound for the development of innovative therapeutics against acute liver injury, addressing a critical unmet need in global healthcare.

## Figures and Tables

**Table 1:** The information on primers.

Primer	Forward (5′→3′)	Reverse (5′→3′)
Caspase-1	CTGGGCAAAGGGAAGACTGTAGATG	ATGATGGCAACGATGGCAGGATAC
GSDMD	GTGAGCCACCCTGCTATTCA	GCAGGCATCCAGGCA ATAGA
NLRP3	GAGCTGGACCTCAGTGACAATGC	AGAACCAATGCGAGATCCTGACAAC
ASC	ATGGTTTGCTGGATGCTCTGTATGG	AAGGAACAAGTTCTTGCAGGTCAGG
GAPDH	ACTCTACCCACGGCAAGTTC	TGGGTTTCCCGTTGATGACC

## Data Availability

All data utilized in this work could be obtained from the corresponding author upon request.

## Supplementary Materials

The following supporting information can be downloaded at: https://www.mdpi.com/article/10.3390/biom15121743/s1, File S1: Western Blotting Figure.

## Abbreviations

ALI	Acute Liver Injury
Andro	Andrographolide
LPS	Lipopolysaccharide
NLRP3	NACHT, LRR and PYD domains-containing protein 3
GSDMD	Gasdermin D
CASPASE1	Cysteinyl aspartate specific proteinase 1
ASC	Apoptosis-associated speck-like protein containing a CARD
AKT1	AKT Serine/Threonine Kinase 1
IL-18	Interleukin-18
IL-6	Interleukin-6
IL-1β	Interleukin-1 Beta
TNF-α	Tumor Necrosis Factor-Alpha
WB	Western Blot
HE	Hematoxylin and Eosin
MAPK14	Mitogen-Activated Protein Kinase 14
CDK1	Cyclin-Dependent Kinase 1
JAK2	Janus Kinase 2
ERBB2	Erb-B2 Receptor Tyrosine Kinase 2
