# Supplementary material for: Integrating Network Pharmacology, Molecular Docking, and Experimental Validation: Andrographolide Attenuates Acute Liver Injury via the NLRP3/Caspase-1/GSDMD-Mediated Pyroptosis Pathway
Source: Biomolecules. 2025 Dec 16;15(12):1743. doi: 10.3390/biom15121743 (PMC12730944; doi:10.3390/biom15121743)

Original Images for Blots （n=3）：

repeat 1 **repeat 2** **repeat 3**

GSDMD

5mg/kgLPS	-	+	+	+	+
Andro(mg/kg)	-	-	25	50	100

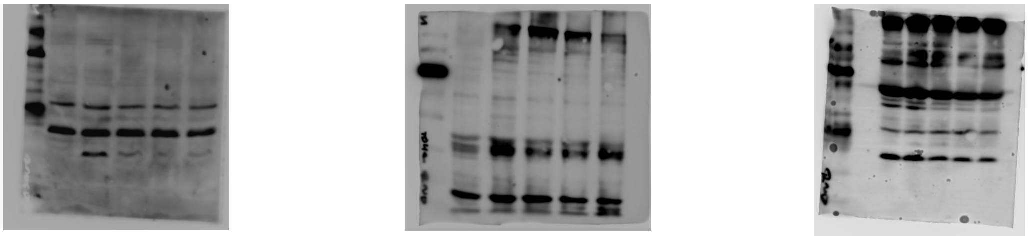

NLRP3

5mg/kgLPS	-	+	+	+	+
Andro(mg/kg)	-	-	25	50	100

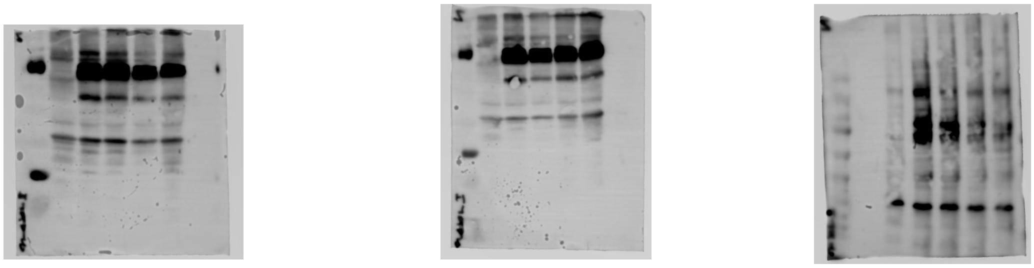

β-tubulin

5mg/kgLPS	-	+	+	+	+
Andro(mg/kg)	-	-	25	50	100

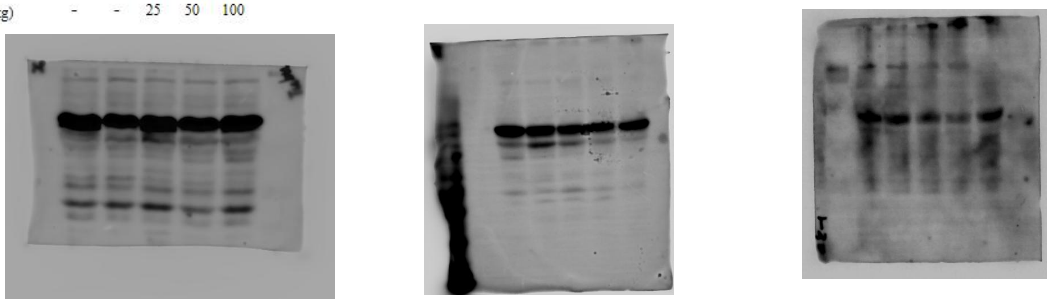

ASC

5mg/kgLPS	-	+	+	+	+
Andro(mg/kg)	-	-	25	50	100

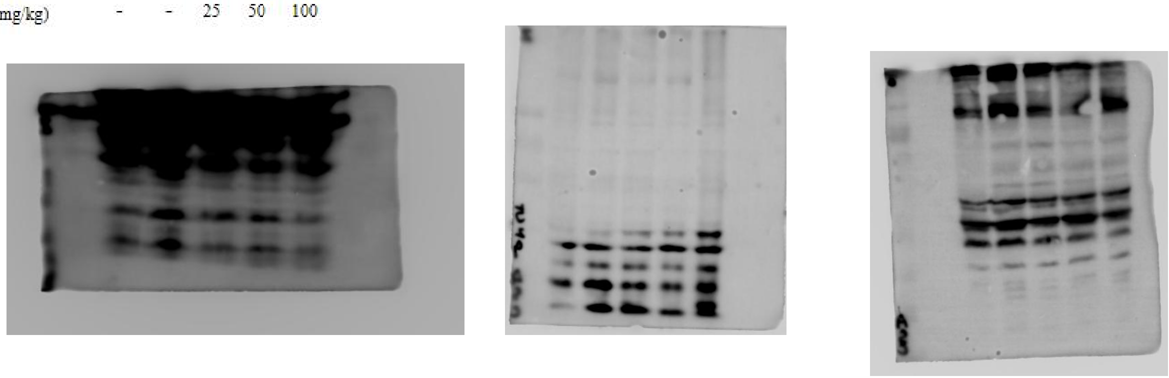

CASPASE-1

5mg/kgLPS	-	+	+	+	+
Andro(mg/kg)	-	-	25	50	100

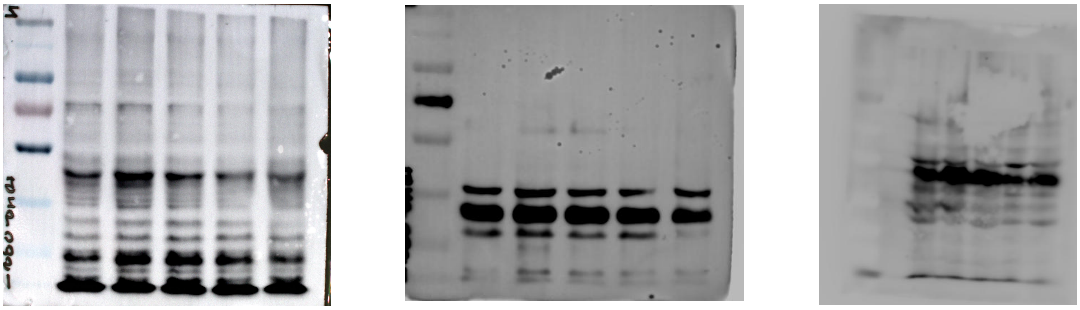

Supplement: Supplementary file 1 [file biomolecules-15-01743-s001.zip › biomolecules-4023927-supplementary.pdf]
